# Contrasting Responses Following Transverse Root Fractures in Two Maxillary Central Incisors: (a) Marginal Breakdown With Superimposed Infection and (b) Transient Internal Surface and Tunnelling Resorption. A Case Report of Management With a 34‐Year 9‐Month Follow‐Up

**DOI:** 10.1111/edt.13049

**Published:** 2025-02-28

**Authors:** Geoffrey S. Heithersay, Lawrence Alvino

**Affiliations:** ^1^ Discipline of Endodontics and Dental Traumatology, Adelaide Dental School The University of Adelaide Adelaide South Australia Australia

## Abstract

An example of contrasting responses in two root fractured teeth in a 16‐year‐old male seriously injured in a hit‐and‐run motor accident is documented over a 34‐year 9‐month period. Extended non‐surgical endodontic management was necessary for one of the root‐fractured central incisors that had developed an infective marginal breakdown, and by contrast, the contralateral incisor with a diagnosis of internal surface resorption and internal tunneling resorption was carefully monitored to ultimate resolution.

## 1 Introduction

Root fractures in permanent teeth are relatively uncommon and have been reported to constitute 0.5%–7% of traumatic dental injuries, with sporting, recreational, vehicular accidents, and physical assaults being the principal causes. The three healing responses to root fractures are (1) hard tissue formation at the fracture site, (2) interposition of fibrous connective tissue, and (3) interposition of periodontal ligament and bone. Nonhealing occurs when there has been pulp necrosis with bacterial invasion within the coronal segment of a root‐fractured tooth, resulting in inflammatory root and bone resorption at the fracture site [1, 2].

There have been reports of marginal breakdown related to luxation injuries, principally intrusive luxations and to a lesser extent lateral luxations [3, 4]. Some cases of marginal breakdown are transitory with uncomplicated regeneration of alveolar bone, while others remain with a loss of alveolar support. However, there do not appear to have been any reports to date of marginal breakdown associated with root‐fractured teeth with luxation of the coronal segment. This includes two studies of 400 root‐fractured teeth [5, 6] and a third study with 534 root‐fractured incisors [7].

In respect to transient internal resorption in root‐fractured teeth, an early report entitled “Fractured root with internal resorption, repair and formation of callus” was published in the first edition of the Journal of Endodontics in 1975 authored by Dr. John Hartness [8]. This clinical report illustrated transient internal resorption at the fracture site in both the coronal and apical segments of a root‐fractured central incisor. Ultimately hard‐tissue repair became evident after 4 years of clinical and radiographic monitoring.

A more detailed analysis was provided in an extensive study of 95 root fractured incisors in 1988 by Andreasen F. M., Andreasen J. O. entitled “Resorption and mineralisation following root fracture of permanent incisors” [9]. The internal resorptions they reported within the root canal system was designated into: (1) internal surface resorption and (2) internal tunnelling resorption. Both types of internal resorption were identified in their study. Of 95 root‐fractured teeth, 37 showed internal surface resorption, while 18 showed internal tunnelling resorption (Ref Table 2 from the study). Both types of resorptions were significantly more frequent after concomitant extrusive luxations compared to concomitant lateral luxations or subluxations. Overall, 59% of teeth in their study developed transient internal resorptions which indicates that they are relatively common in root‐fractured teeth. The study demonstrated that internal surface resorptions became evident radiographically within 3 weeks–1 year and resolved without treatment over an unstated period. Internal tunnelling resorptions developed 3 weeks–5 years after injury and were reported to have partially or completely healed between 6 months and 5 years.

## 2 Case Report

Andrew, a 16‐year‐old male, was riding his bicycle to school when he suffered a serious accident after being hit by the driver of a motor vehicle who did not stop and left the victim lying in an unconscious state on the roadway. Concurrently, he suffered injuries to his head, face, and maxillary central incisors. Fortunately, a neighbour heard the loud noise of the accident and came to the seriously injured young male's aid and immediately called an ambulance, which arrived within 10 min. He was taken to a nearby state hospital's casualty department in an unconscious state and admitted for close observation, where he remained for 3 days. During that time, he regained consciousness, and his extra‐oral facial wound extending from his nose to his upper lip was sutured. No treatment was carried out for the extruded, root‐fractured maxillary right central incisor; although on discharge from the hospital, he was advised to seek dental management from his school dental clinic as soon as possible. However, when seen by a school clinic dentist shortly after discharge, Andrew had significant facial swelling and could only open his mouth approximately 6 mm, which precluded a satisfactory assessment of his dental injuries.

**FIGURE 1:**
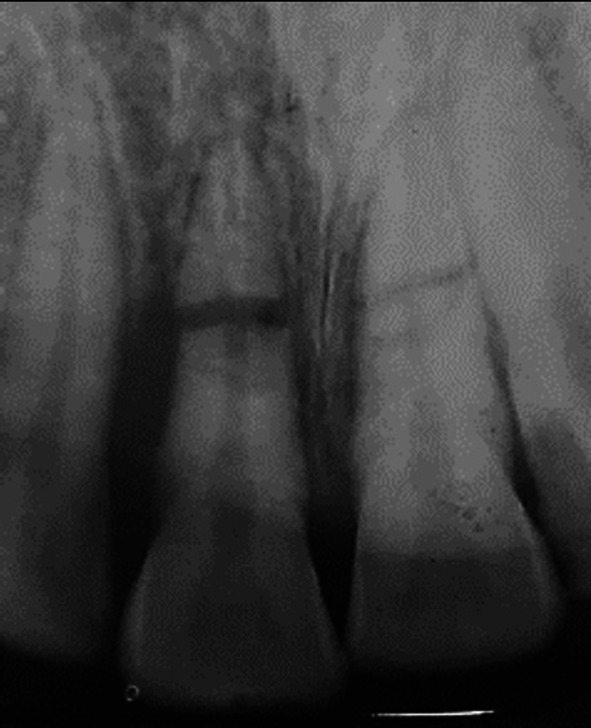
Radiograph taken 10 days post‐trauma showing root fractures in both maxillary central incisors with 11 extrusively luxated 3–4 mm and 21 relatively undisplaced. A radiolucency on the distal aspect of 11 indicates early marginal bone loss.

When seen 7 days later, 10 days after the accident, his facial swelling had resolved sufficiently for the school dentist to be able to examine his dental injuries. He recorded that the facial wounds were healing satisfactorily, while intra‐orally, the right central incisor had clearly been extrusively luxated approximately 2 mm and was quite mobile. A radiograph taken at that time (Figure 1) provided evidence that both maxillary central incisors had suffered transverse root fractures, with the left central incisor (21) relatively undisplaced, while the right central incisor (11) was extrusively luxated. In addition, a radiolucency on the distal aspect of 11, extending from the level of the transverse fracture to the cervical region, indicated early marginal bone loss. The dentist repositioned 11 with some difficulty and then placed a composite and orthodontic wire flexible splint. Pulp sensibility testing proved negative for both maxillary central incisors. Oral hygiene instructions were provided, and a supporting antibiotic cover of Phenethicillin was prescribed. Andrew was then referred for endodontic management by the principal author (G.S.H.).

**FIGURE 2:**
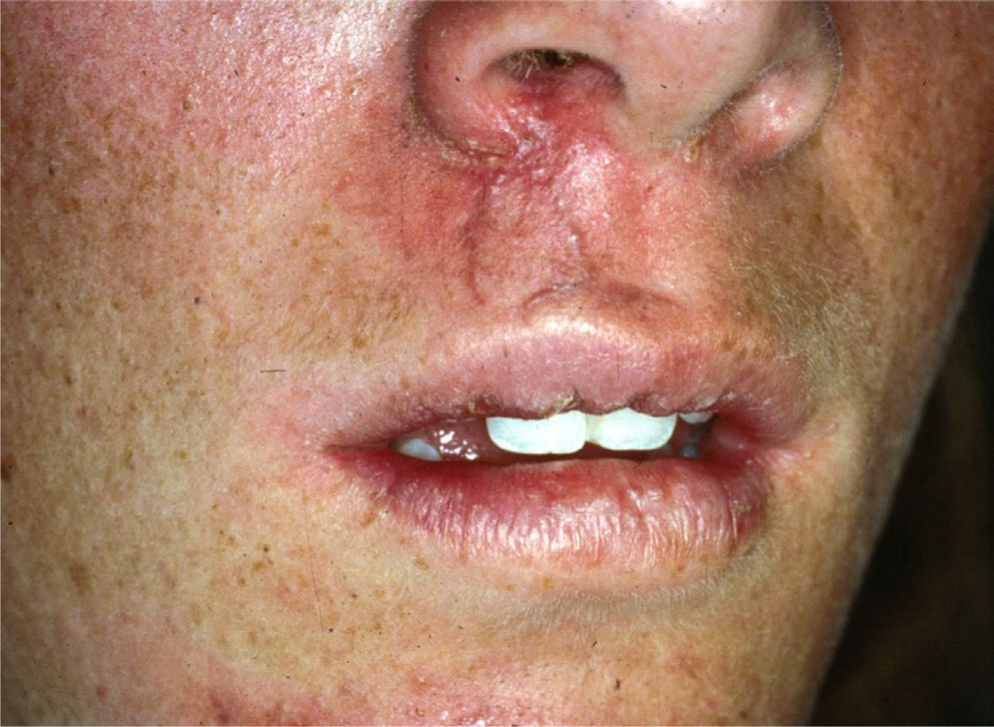
Facial view of the patient 24 days post‐trauma showing the healing lacerated wounds extending from the nose to the vermillion border of the upper lip.

**FIGURE 3:**
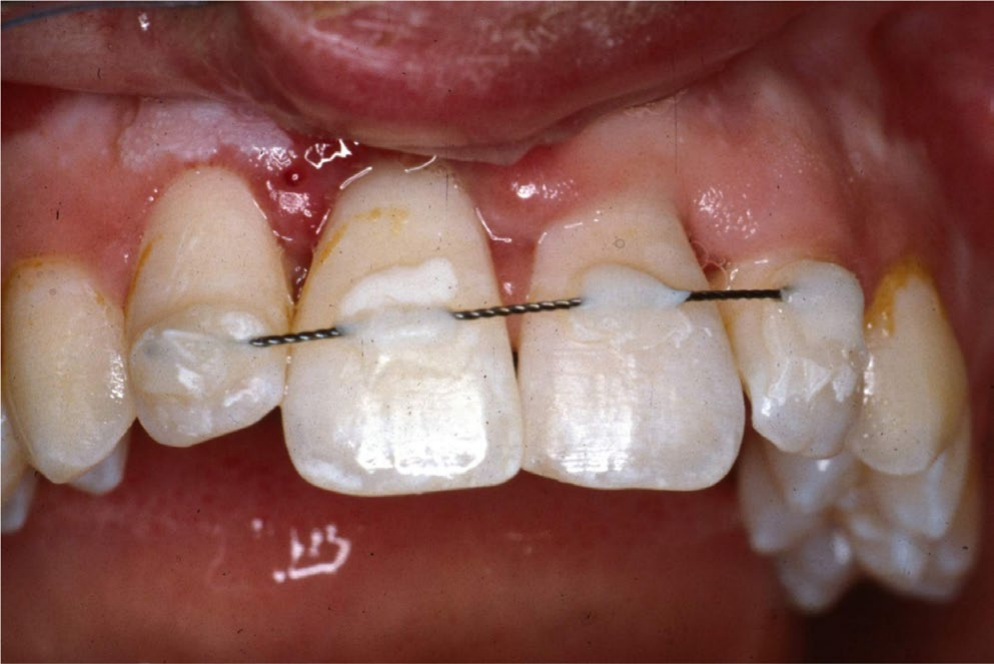
Appearance of splinted teeth 24 days post‐trauma showing 11 to have been repositioned into a more favourable position, but gingival inflammation is evident, and a slight colour change is evident in the gingival third of the crown of 21.

**FIGURE 4:**
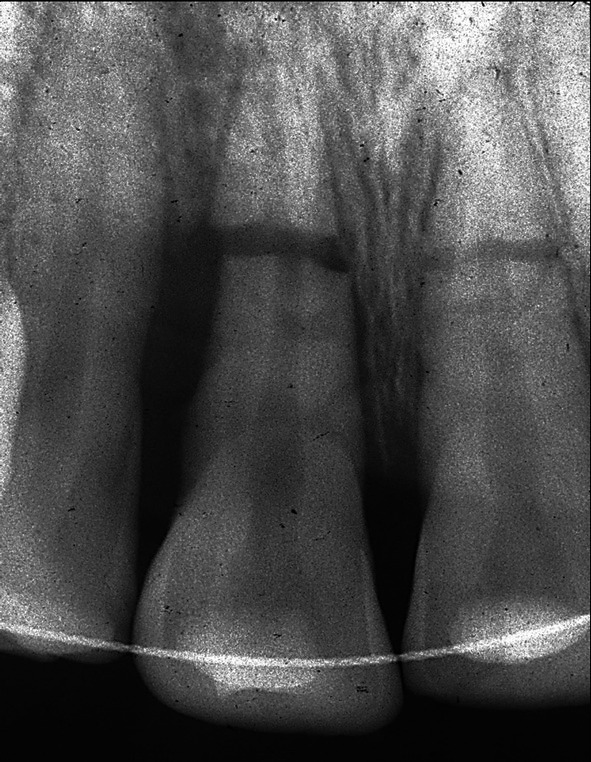
Radiograph showing evidence of marginal bone loss on the distal aspect of 11.

When the normally healthy Andrew was examined 14 days later, 24 days post‐trauma, the previously sutured extra‐oral lacerations which extended from the base of the nose to the vermilion border of the upper lip showed satisfactory healing (Figure 2). Intra‐orally the splint was still in place, but 11 still appeared to be in a slightly extruded position and the associated gingival tissues showed inflammatory changes (Figure 3). A slight colour change was noted in the gingival third of the tooth crown of 21. A radiograph of the teeth taken at that time is shown in Figure 4. Of significance was the further radiographic evidence of marginal bone loss on the distal aspect of 11. There was no evidence of pathosis related to the relatively un‐displaced 21. As pulp sensibility testing of 11 with CO_2_ Odontotest proved negative, a diagnosis of “traumatically induced marginal breakdown with superimposed infection from a necrotic pulp in the coronal segment of the root fractured 11” was deemed appropriate. The complexity of the presenting trauma‐related clinical issues and the proposed treatment plan were detailed to Andrew and his mother and accepted.

**FIGURE 5:**
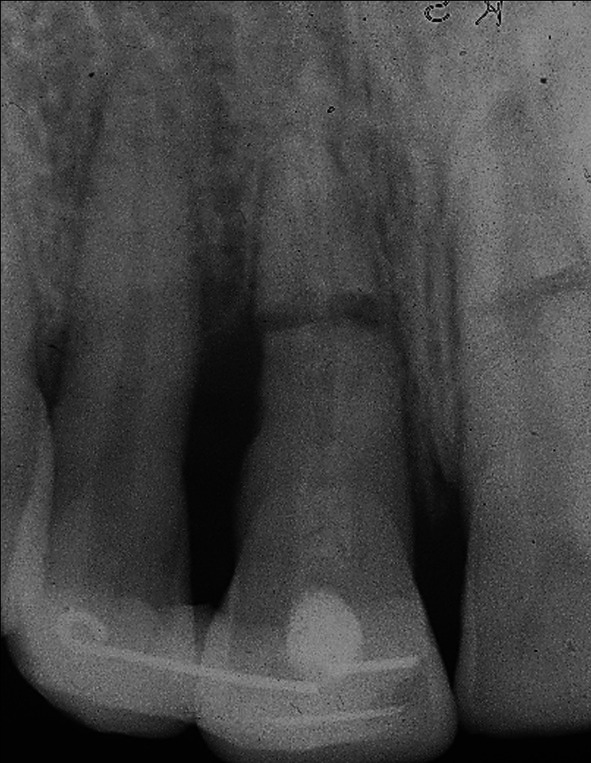
Radiograph taken at the time of the replacement of the original flexible splint with a fixed composite splint.

**FIGURE 6:**
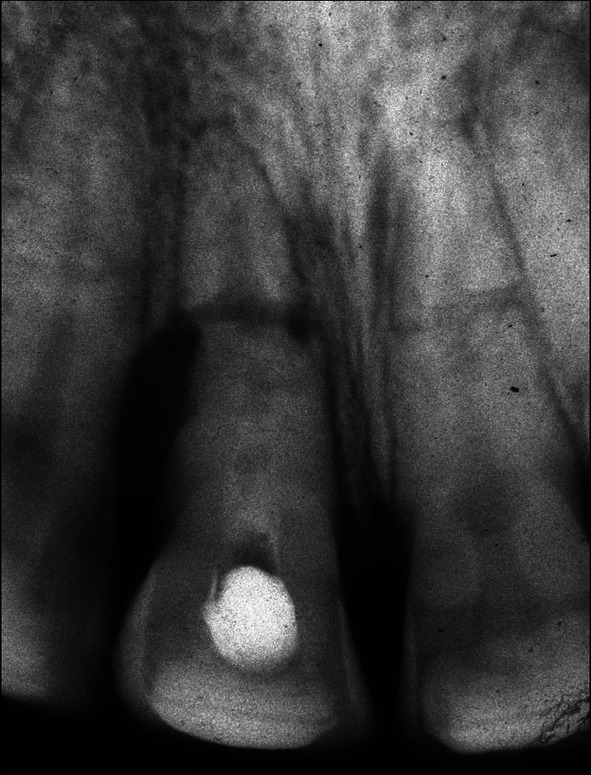
Radiograph taken 7 months posttreatment of 11 shows no evidence of bone regeneration of the marginal bone loss on its distal aspect. Transient Internal surface resorption can be observed in the crown of 21.

The diagnosis of pulp necrosis in the coronal segment of 11 was confirmed clinically when non‐surgical root canal therapy was carried out without the need for local anaesthesia. Root canal debridement of the coronal segment of the root canal involved instrumentation with Hedström files coupled with the sequential irrigation with 8% EDTA‐C solution and 0.9% sodium hypochlorite, which was further activated by ultrasonication. After a final rinse with EDTA‐C solution, the canal was dried with paper points, and then a 50/50 mix of Ledermix paste (Lederle Laboratories, Wolfratshausen Germany) and Pulpdent Paste (Pulpdent Corporation, Watertown MA USA) was spun into the canal prior to the placement of a double coronal seal of Cavit (3 M ESPE, St Paul Minnesota USA) and a glass ionomer cement (Ketac GC Corp, Tokyo Japan). The root canal dressing was replaced after 3 weeks and again 2 months after the commencement of endodontic therapy. At that time, the original flexible splint was removed from 21 to 22, but as 11 then showed a Miller grade 3 mobility, the incisal edge was adjusted to reduce any occlusal interference, and a rigid composite splint, which incorporated part of the original splint, was placed. Supporting antibiotic (amoxycillin V) was also prescribed. A radiograph taken at that time is shown in Figure 5. A dressing change using the same medicament combination was carried out on four separate occasions, which were determined by clinical signs and symptoms. Seven months posttreatment, there had been clinical signs of improvement of the tissues on the distal aspect of 11, but a radiograph taken at that time showed no indication of marginal bone regeneration (Figure 6). Of further significance was the first radiographic evidence of internal surface resorption in 21. One month later, the rigid splint was removed, and the root canal was then dressed with the calcium hydroxide‐based Pulpdent Paste with the aim of inducing some hard tissue at the junction of the coronal segment with the healing fracture site.

**FIGURE 7:**
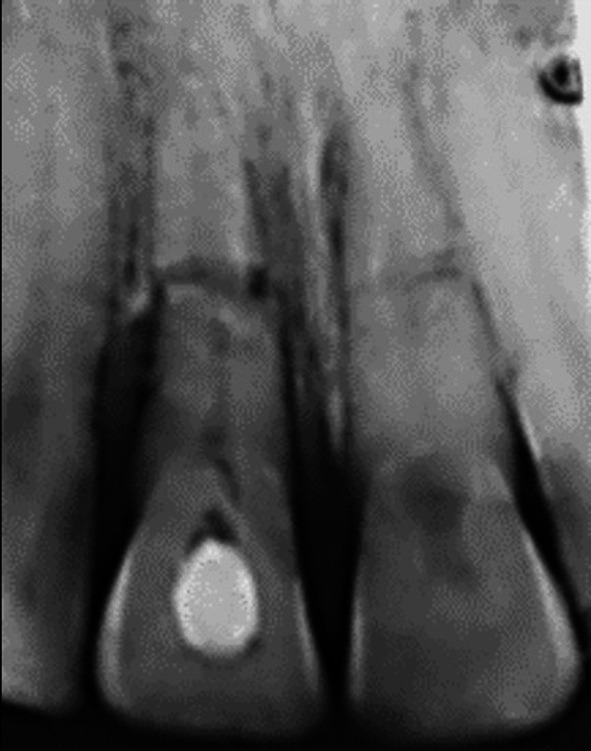
Radiograph taken 10 months posttreatment shows early evidence of marginal bone regeneration in respect to11 while the transient internal surface resorption in 21 has extended coronally.

**FIGURE 8:**
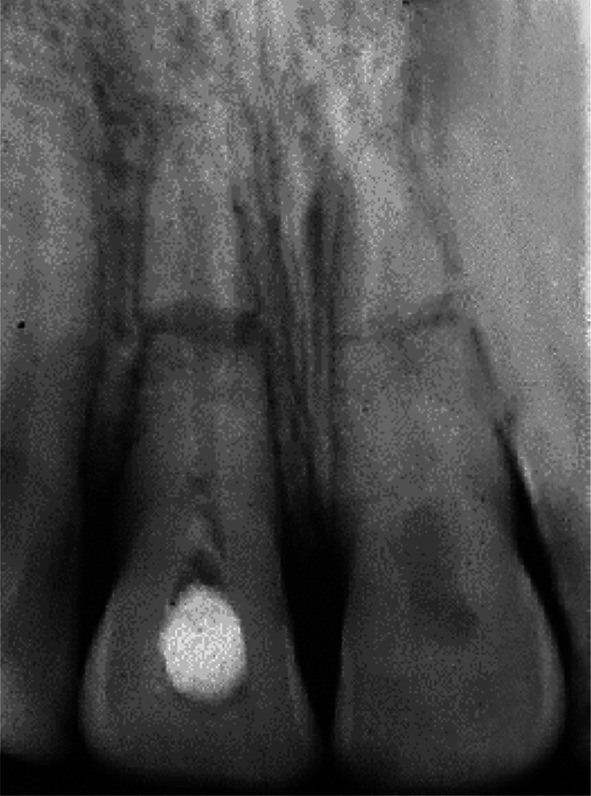
A radiographic review 1‐year 4‐weeks posttreatment shows evidence of significant marginal bone regeneration on the distal aspect of 11, while the transient internal surface resorption in 21 has extended further into the crown.

**FIGURE 9:**
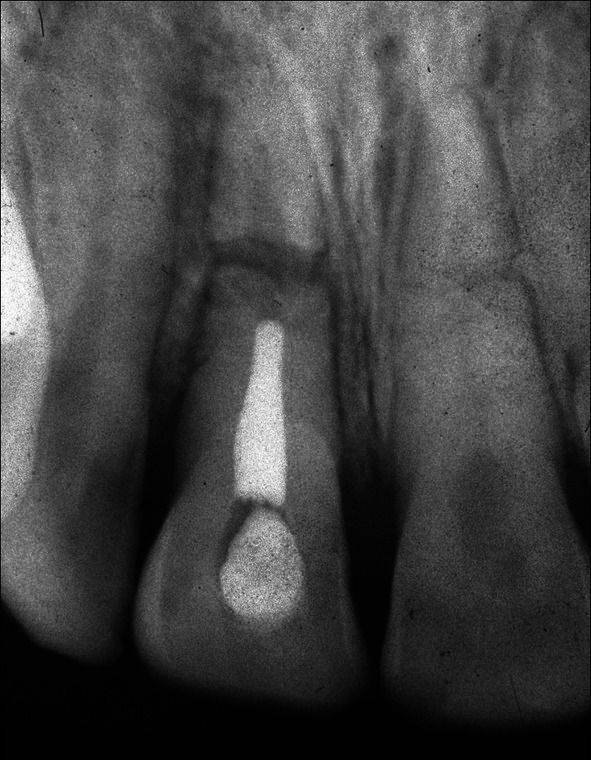
Post root filling radiograph of 11 with a temporary seal with Cavit W prior to bleaching.

A further review 10 months posttreatment showed encouraging signs of some marginal bone regeneration on the distal aspect of 11, while the internal resorption in 21 had extended coronally (Figure 7). When checked again at 1‐year 4‐weeks posttreatment, there was radiographic evidence of further marginal bone regeneration and a favourable healing response at the fracture site, as shown in Figure 8. Furthermore, the image of the internal surface resorption noted earlier in 21 had increased in size, indicative of the internal resorptive process extending further into the crown. At this stage, the root canal of the coronal segment of 11 was able to be root‐filled with gutta‐percha and AH26 to a position where hard tissue had been apically deposited. A post‐root filling radiograph is shown in Figure 9 with the coronal access cavity temporally filled with Cavit‐W prior to bleaching. After the colour had been restored, a coronal seal of the glass ionomer cement Ketac was then placed.

**FIGURE 10:**
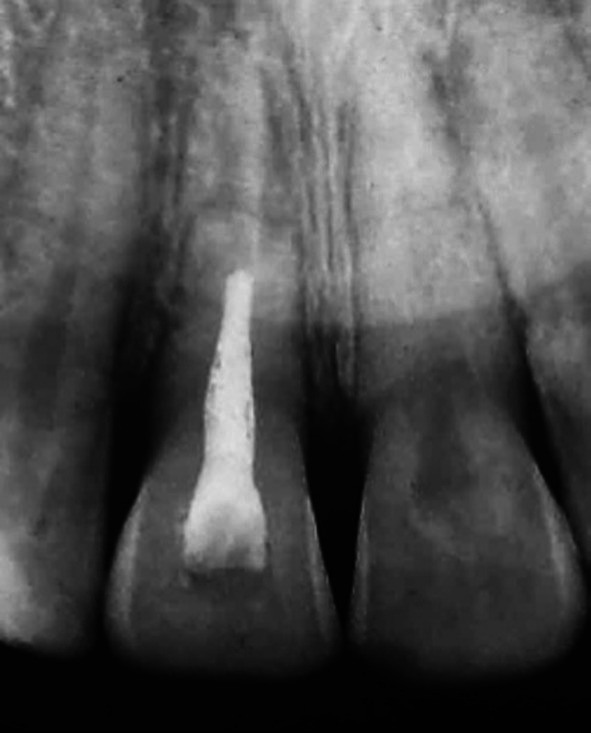
A review 1‐year 7‐months post‐treatment shows radiographic evidence of excellent healing in respect to 11, while the transient internal surface resorption in 21 shows signs of tunneling resorption.

**FIGURE 11:**
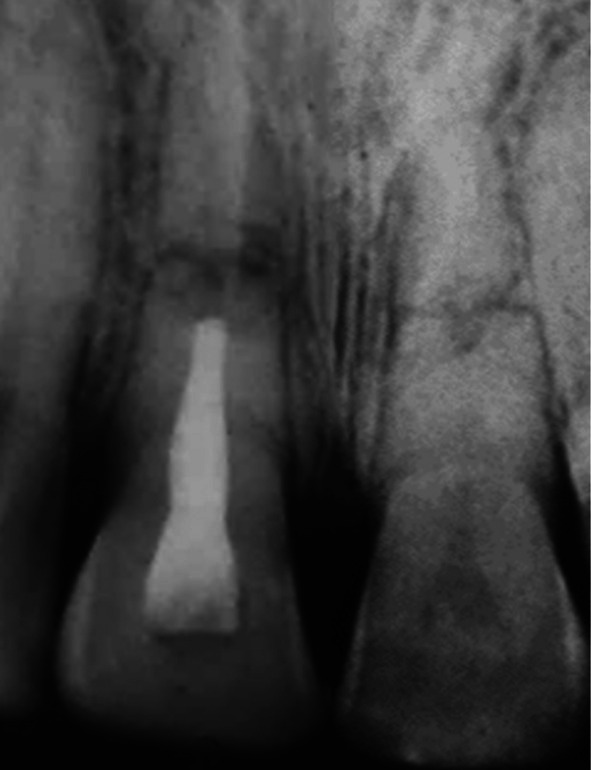
A further review 2 years and 5 months posttreatment shows radiographic evidence of some calcification within the transient internal surface resorptive process indicative of a healing response.

Andrew was reviewed again 6 months later (1‐year 7‐months posttreatment) when there was radiographic evidence of excellent healing in respect to 11, while the internal surface resorption in 21 appeared to be undergoing internal tunneling resorption extending into the coronal aspect of the tooth (Figure 10). A further check 9 months later, 2‐years 5‐months posttreatment (Figure 11) indicated that the resorption in 21 was undergoing a healing phase, with some calcification evident within the internal surface and tunneling resorptive processes. There was also evidence of satisfactory healing at both fracture sites.

**FIGURE 12:**
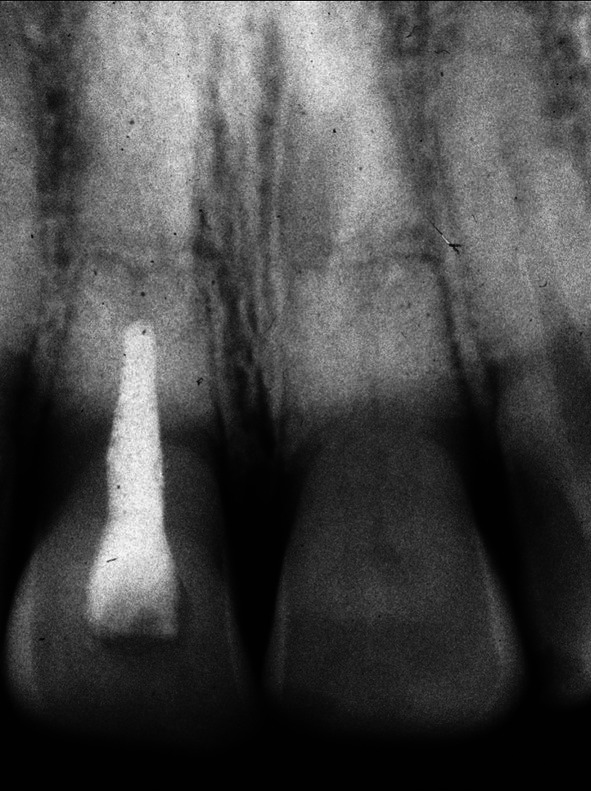
Radiograph taken at 9‐years 10‐months posttreatment shows evidence of healing of the root fractured incisors with the interposition of bone and periodontal ligament lining both fractured segments. Long‐term healing of the transient internal surface and tunneling resorptive defect in 21 is evident.

**FIGURE 13:**
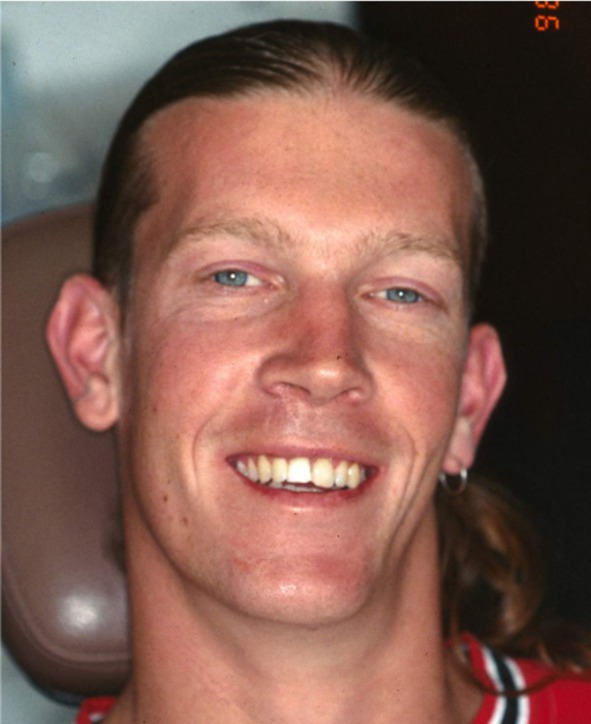
Photograph of Andrew at 9‐years 10‐months posttreatment shows a satisfactory aesthetic result in respect to his traumatized maxillary central incisors.

Andrew was then reviewed on a three‐yearly basis, with satisfactory healing observed on each occasion. A review radiograph taken in 1998—9‐years 10‐months post‐injury is—shown in Figure 12. This shows clear evidence of long‐term healing in respect to the treated 11 and the untreated 21. The fractures in both teeth show radiographic evidence of healing, with the interposition of bone and a periodontal ligament lining both fractured segments. The longer‐term healing of the internal surface resorptive defect is evidenced radiographically by the significant degree of root canal calcification in 21. However, a fine residual canal could be observed. A photograph of Andrew taken at that time, shown in Figure 13, reveals a satisfactory aesthetic result.

**FIGURE 14:**
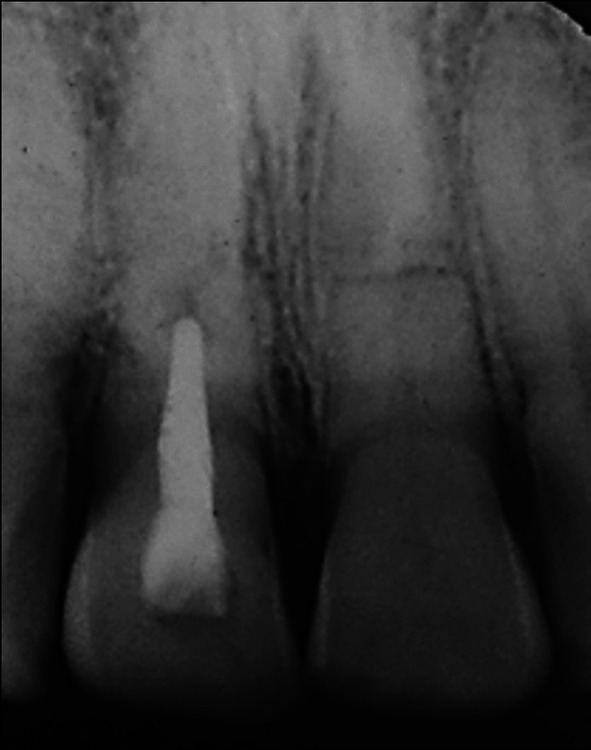
Radiograph taken 13 years posttreatment shows evidence of satisfactory healing at the fracture site of both central incisors.

**FIGURE 15:**
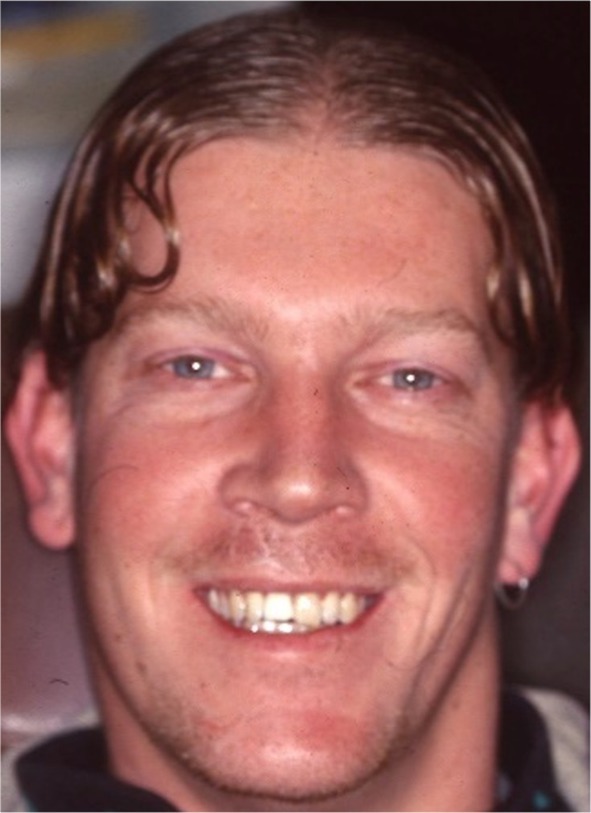
A photograph shows a satisfactory aesthetic appearance; however, there is slight calcific‐related discolouration in 21.

A further review in 2002—13 years post‐injuries showed satisfactory progress in both central incisors with no radiographic or clinical evidence of periradicular or periapical pathosis (Figure 14). The maxillary left central incisor shows an increase in the degree of calcification‐induced discoloration as shown in Figure 15.

**FIGURE 16:**
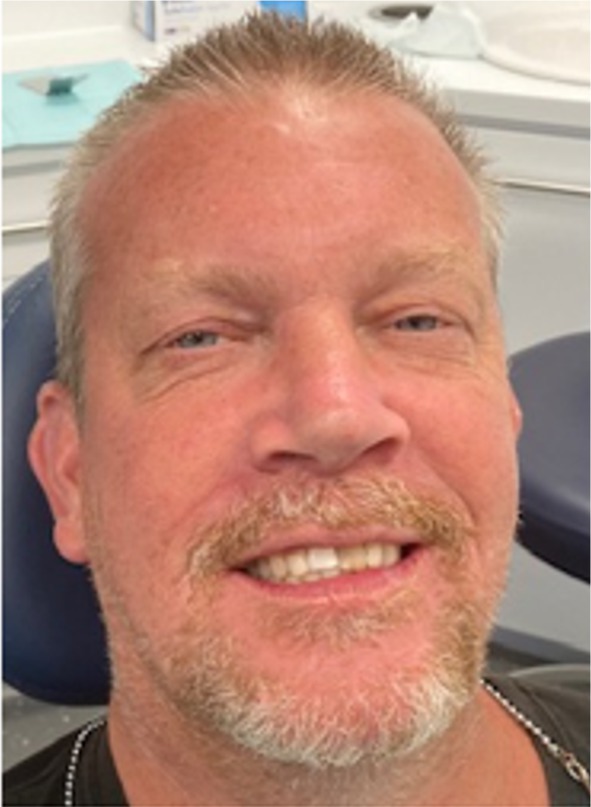
Facial view of Andrew at 34‐years 9‐months posttreatment.

**FIGURE 17:**
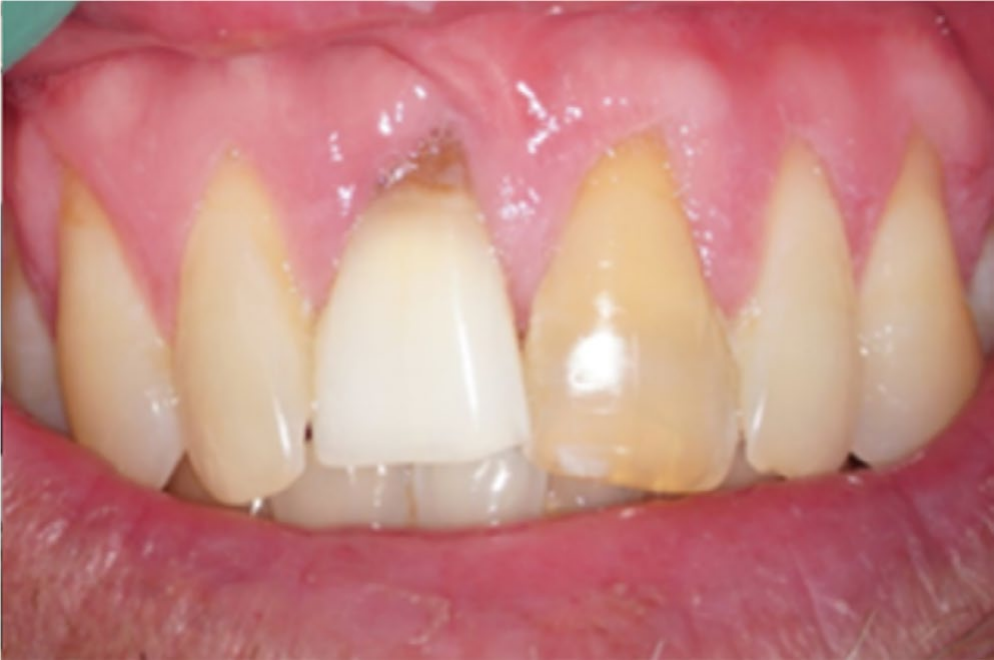
Appearance of both root‐fractured maxillary central incisors showing long‐term calcification‐induced discolouration of the crown of 21 and some degree of clefting of the gingival tissues related to both incisors.

**FIGURE 18:**
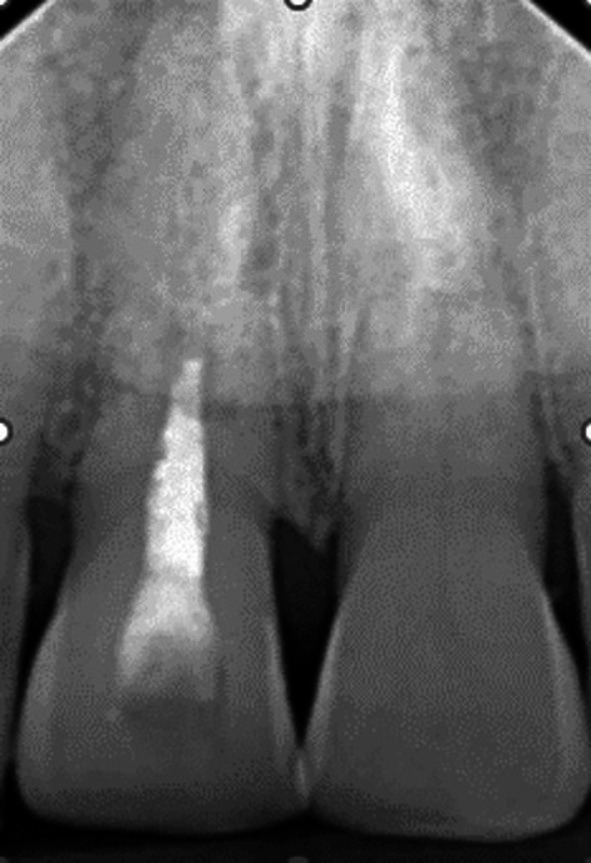
Radiograph taken at 34‐years 9‐months posttreatment shows no evidence of peri‐radicular pathosis.

In 2024, 34‐years 9‐months after his injuries, Andrew, aged 49, married with two teenage children, agreed to be reviewed to assess the long‐term outcome of his management. Photographic images taken at that time are shown in Figures 16 and 17. The discolouration of 21 had intensified over the ensuing years, but due to Andrew's low lip line, it was not of aesthetic concern to him. There were localized gingival clefts associated with both maxillary central incisors, but probing depths were within normal limits. Radiographically, both root fractured central incisors were assessed as being free of pathosis (Figure 18). Radiographically, a fine residual canal was still in 21.

**FIGURE 19:**
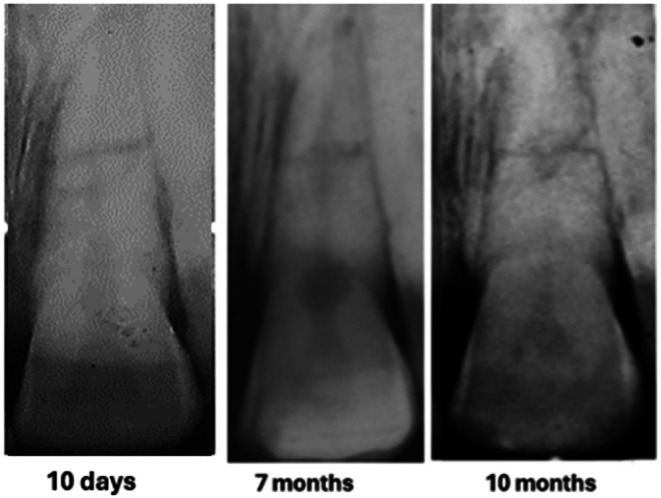
Radiographic appearance of the development of transient internal surface resorption of 21 from 28 days post‐trauma to its first evidence at 7 months. At 10 months, extension into the crown was evident.

**FIGURE 20:**
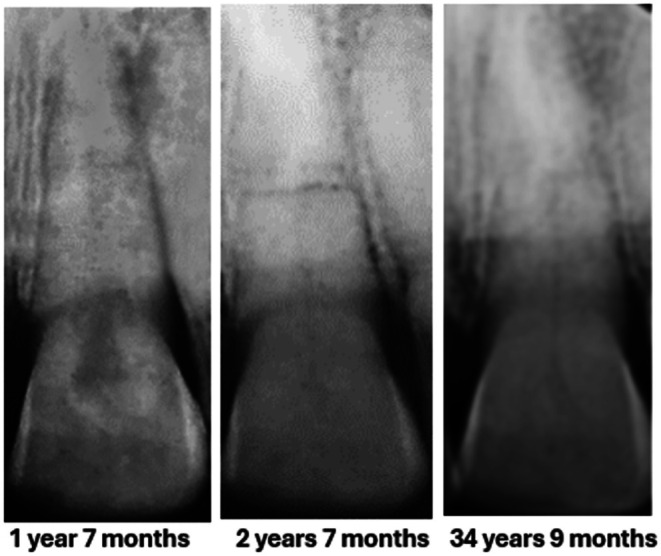
Radiographic appearance of the development of tunneling resorption at 1 year 7 months followed by progressive calcification by 2 years 7 months and the long term (34 years 9 months) appearance of 21 with a residual fine root canal indicative of calcific root canal stenosis.

The radiographic appearance of the development and progression of the transient internal surface resorption and internal tunnelling resorption to its ultimate healing is shown in Figures 19 and 20.

## 3 Discussion

When Andrew was seen in the emergency department of a major hospital, management correctly focused on his neurological state, and his dental injuries were not addressed. Immediately on discharge from hospital, he sought dental management, but because of facial swelling and restricted jaw opening, it was a further 7–10 days post‐trauma before repositioning of the extrusively luxated coronal segment of the root fractured 11 and flexible splinting could be carried out. The difficulty encountered by Andrew's dentist in fully repositioning 11 was likely due to the organizing state of the blood clot in the socket and/or to the presence of small fractured alveolar bone fragments.

The optimal management for root fractured teeth as detailed in the IADT 2020 Guidelines for the management of dental injuries states “If displaced, the coronal fragment should be repositioned as soon as possible” [10]. This recommendation contrasts with an earlier major study into the effect of various treatment factors in intra‐alveolar root fractures, including treatment delay. The authors had concluded that “a certain treatment delay (a few days) appears not to result in inferior healing” [6].

The significant complication of marginal bone loss with superimposed infection could well be considered to be due to the extent of the trauma inflicted not only to the maxillary right central incisor but also to the supporting alveolar bone, probably causing a small alveolar fracture. There would also be damage to the periodontal and associated soft tissues. Had it been possible for the early repositioning of all these hard and soft tissues as recommended by the IADT guidelines less complicated healing may have been possible.

While the initial flexible orthodontic wire and composite splint was appropriate to aid in periodontal and alveolar bone healing, the decision to create a rigid splint after its removal from 21 was deemed appropriate due to the significant mobility of 11. Management of the infective marginal breakdown was aided by long‐term splinting and intra‐canal debridement and dressing with Pulpdent paste, initially in combination with Ledermix paste and later alone. Some regeneration of alveolar bone became subtly evident radiographically after 7 months and was complete by 12 months. Healing at the fracture site was by the interposition of bone and periodontal ligament.

Radiographically, the internal surface resorptive process was first noted 7 months post‐injury, increasing in size into the tooth crown, then evolving into internal tunneling resorption by 1 year and 7 months, before slowly resolving with progressive calcification by 2 years and 7 months. In a longer‐term radiographic examination at 34 years and 9 months, a fine residual root canal could still be observed.

The discolouration associated with the coronal calcification in 21 was first noted after 6 years and progressively intensified by the long‐term evaluation. As there was no radiographic or clinical evidence of pathoses in either of the root fractured teeth at the long‐term examination, the prognosis for their permanent retention was considered to be favorable.

The psychological effect of dental trauma on young patients has been well documented and considered to be dependent on the magnitude of the injuries and how they have been managed [11]. Empathy between the clinician and the patient and family is essential for a favourable outcome. The authors submit that these criteria have been met in the overall management of Andrew's near‐death trauma involving severe head, orofacial, and dental injuries. It has been a pleasure and a privilege to document over an extended period the management of this remarkably resilient patient.

## 4 Conclusion

A diagnosis of marginal bone loss or transient internal resorptions, either in the form of internal surface resorption or internal tunnelling resorption in the root‐fractured teeth, highlights the need for the recognition of the underlying tissue responses and their management, either by non‐surgical root canal treatment or careful monitoring. The aim of all management strategies should be the lifetime retention of root‐fractured teeth.

## Author Contributions

Geoffrey Heithersay was responsible for the endodontic management and recording of the patients dental injuries and was the principal author. Lawrence Alvino contributed to the long term clinical and radiographic examination and to the editing of the report.

## Disclosure

This case report has been written according to the Preferred Reporting Items for Case reports in Endodontics (PRICE) 2020 guidelines (Data S1).

## Conflicts of Interest

The authors declare no conflicts of interest.

## Supporting information


Data S1.

## Data Availability

The data that support the findings of this study are available from the corresponding author upon reasonable request.
