# Supplementary material for: Contrasting Responses Following Transverse Root Fractures in Two Maxillary Central Incisors: (a) Marginal Breakdown With Superimposed Infection and (b) Transient Internal Surface and Tunnelling Resorption. A Case Report of Management With a 34‐Year 9‐Month Follow‐Up
Source: Dent Traumatol. 2025 Feb 28;41(4):476–83. doi: 10.1111/edt.13049 (PMC12260119; doi:10.1111/edt.13049)
Supplement: Supplementary file 1 — Data S1. [file EDT-41-476-s001.zip › PRICE Flowchart.docx]

**PRICE 2020 Flowchart**

***From: Nagendrababu V, Chong BS, McCabe P, Shah PK, Priya E, Jayaraman J, Pulikkotil SJ, Setzer FC, Sunde PT, Dummer PMH (2020) PRICE 2020 Guidelines for reporting case reports in Endodontics: A consensus-based development. *International Endodontic Journal* doi: 10.1111/iej.13285.**

**For further details visit:** [**http://pride-endodonticguidelines.org/price/**](http://pride-endodonticguidelines.org/price/)
